# *QuickStats:* Percentage* of Families That Did Not Get Needed Medical Care Because of Cost,^†^ by Poverty Status^§^ — National Health Interview Survey, United States, 2013 and 2018

**DOI:** 10.15585/mmwr.mm6923a4

**Published:** 2020-06-12

**Figure:**
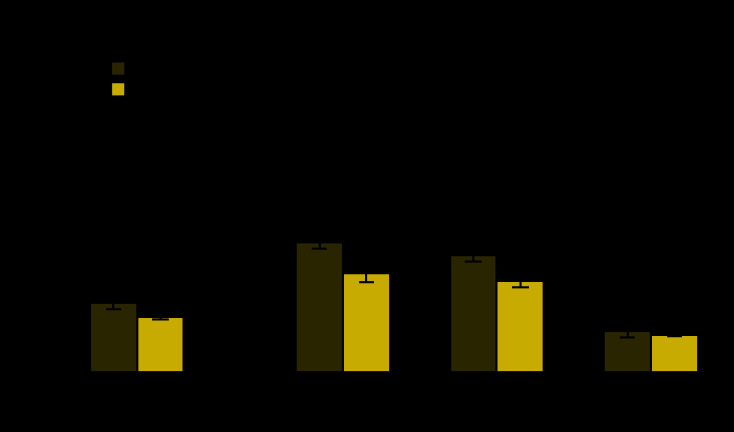
The percentage of all families that did not get needed medical care because of cost in the past 12 months decreased from 12.1% in 2013 to 9.7% 2018. From 2013 to 2018, the percentage of poor families that did not get medical care decreased (22.7% to 17.3%) as did the percentage of near-poor families (20.4% to 16.0%); no significant change occurred for not-poor families (7.1% and 6.6%). In 2013 and 2018, the percentage of families that did not get needed medical care because of cost was lowest among the not poor.

**Source:** National Health Interview Survey, 2013 and 2018 data. https://www.cdc.gov/nchs/nhis.htm.

